# In Vitro Biophysical and Biological Characterization of Lipid Nanoparticles Co-Encapsulating Oncosuppressors miR-199b-5p and miR-204-5p as Potentiators of Target Therapy in Metastatic Melanoma

**DOI:** 10.3390/ijms21061930

**Published:** 2020-03-12

**Authors:** Luigi Fattore, Virginia Campani, Ciro Francesco Ruggiero, Valentina Salvati, Domenico Liguoro, Lorena Scotti, Gerardo Botti, Paolo Antonio Ascierto, Rita Mancini, Giuseppe De Rosa, Gennaro Ciliberto

**Affiliations:** 1 Istituto Nazionale Tumori IRCCS, "Fondazione G. Pascale", 80131 Naples, Italy; l.fattore@istitutotumori.na.it (L.F.); g.botti@istitutotumori.na.it (G.B.); paolo.ascierto@gmail.com (P.A.A.); 2 Department of Pharmacy, University of Naples Federico II, 80131 Naples, Italy; virginia.campani@unina.it (V.C.); lorena.scotti@unina.it (L.S.); gderosa@unina.it (G.D.R.); 3 IRCCS, Istituto Nazionale Tumori “Regina Elena”, Via Elio Chianesi 53, 00144 Rome, Italy; cirofrancescoruggier@libero.it (C.F.R.); salvati.sv@gmail.com (V.S.); 4 Department of Molecular and Clinical Medicine, University of Roma “Sapienza”, 00185 Rome, Italy; do.liguoro@gmail.com (D.L.); rita.mancini@uniroma1.it (R.M.)

**Keywords:** Melanoma, nanoparticles, miRNAs, therapeutics, drug resistance

## Abstract

Uncontrolled MAPK signaling is the main oncogenic driver in metastatic melanomas bearing mutations in BRAF kinase. These tumors are currently treated with the combination of BRAF/MEK inhibitors (MAPKi), but this therapy is plagued by drug resistance. In this context we recently discovered that several microRNAs are involved in the development of drug resistance. In particular miR-204-5p and miR-199b-5p were found to function as antagonists of resistance because their enforced overexpression is able to inhibit melanoma cell growth in vitro either alone or in combination with MAPKi. However, the use of miRNAs in therapy is hampered by their rapid degradation in serum and biological fluids, as well as by the poor intracellular uptake. Here, we developed lipid nanoparticles (LNPs) encapsulating miR-204-5p, miR-199b-5p individually or in combination. We obtained LNPs with mean diameters < 200 nm and high miRNA encapsulation efficiency. These formulations were tested in vitro on several melanoma cell lines sensitive to MAPKi or rendered drug resistant. Our results show that LNPs encapsulating combinations of the two oncosuppressor miRNAs are highly efficient in impairing melanoma cell proliferation and viability, affect key signaling pathways involved in melanoma cell survival, and potentiate the efficacy of drugs inhibiting BRAF and MEK. These results warrant further assessment of the anti-tumor efficacy of oncosuppressor miRNAs encapsulating LNPs in in vivo tumor models.

## 1. Introduction

Melanoma is the most severe malignant form of skin cancer and is characterized by high propensity to spread to other parts of the body [1]. In the last years, targeted therapy and immunotherapy have revolutionized treatment of metastatic melanoma [2,3]. Targeted therapy was conceived following the discovery that about 50% of patients bear activating mutations in the BRAF oncogene in their tumors [4]. For these patients BRAF and MEK inhibitors in combination have become the gold standard of first-line therapy because this approach showed superiority over monotherapy with BRAF inhibitor to contain the development of drug resistance and to improve time to recurrence and overall survival [5,6,7]. For BRAF wild-type melanomas, the therapeutic approach is the use of first-line therapy immune checkpoint inhibitors (ICI) with antibodies against PD-1/PD-L1, either alone or in combination with anti CTLA4 antibodies [8,9,10]. Although both targeted therapy and immunotherapy have improved metastatic melanoma patients’ survival, drug resistance, either innate or acquired, severely limits their efficacy [11,12,13].

Our group has been focusing over the last years on the study of non-genetic mechanisms at the basis of drug resistance to MAPKi in BRAF-mutated melanomas [14,15,16]. For example, we have demonstrated that a member of the EGFR family, the ErbB3 receptor, is activated upon exposure to MAPKi by the autocrine production of its ligand neuregulin-1 [17], and that circulating tumor cells (CTCs) derived from melanoma patients undergo up-regulation of ErbB3 phosphorylation in vivo shortly after initiation of therapy [18].

We have also demonstrated that inhibition of this receptor using neutralizing antibodies is able to impair the establishment of resistance to MAPKi both in vitro and in vivo [19].

Moving forward, in order to study other potential non mutational modulators of resistance to MAPKi in melanoma, we have investigated the role of microRNAs [20]. Towards this goal we first identified a novel oncosuppressor miRNA, miR-579-3p, as a novel master regulator of MAPKi resistance in BRAF-mutant melanoma patients [21]. Thereafter we carried out an intensive analysis of the entire miRnome before and after the establishment of in vitro resistance to BRAF inhibition [22]. This led to the identification of a set of 29 deregulated miRNAs which could be divided in miRNAs facilitators or antagonists of drug resistance, and whose expression allows to distinguish drug-sensitive from drug-resistant cells [22]. In particular, we characterized in greater detail the biological activity of four additional miRNAs involved in the establishment of drug resistance, the two oncosuppressors miR-204-5p and miR-199b-5p and the two onco-miRs miR-4443 and miR-4488 [22]. Through a combination of transfection studies or miRNA mimics or miRNA antagonists, we showed that these four microRNAs play an important role in drug resistance and affect in a positive or negative manner the efficacy of MAPKi based therapy [22]. These findings open the possibility to develop miRNA-based therapeutics in order to potentiate the activity of MAPKi and to impair the development of drug resistance.

In this paper we focused our attention on oncosuppressors miR-204-5p and miR-199b-5p and their combinations. The use of these molecules offers the possibility to target simultaneous multiple pathways involved in tumor development, progression and drug resistance, providing the opportunity to develop new powerful drugs for the therapy of cancer [23]. However, the use of miRNA-based drugs is hampered by the rapid degradation by nucleases and the poor and unspecific cellular uptake [12,24]. Nanotechnology can overcome these biopharmaceutical issues, because of its potential to preserve RNA stability and to enhance intracellular uptake [25].

Among nanotechnologies used for RNA delivery, lipid nanoparticles (LNPs) characterized by cationic lipids were largely investigated in vitro, while aggregation in serum limits their in vivo use. The introduction of ionizable lipid make it possible to neutralize the surface net charge of lipid nanovectors, making them more stable for application in presence of serum and in vivo [26]. These lipid nanoparticles have shown efficient RNA delivery and safety in clinical trials [27]. Indeed, recently, the first drug based on RNA and containing LNP (ONPATTRO) received the approval for the market. LNPs must be considered at the moment the most mature and safe approach to support the development of RNA-based therapy.

The aim of this work is to propose lipid nanoparticles (LNPs) encapsulating miR-204-5p, miR-199b-5p or a mix of both, as base for a novel approach in the treatment of metastatic melanoma, directed to overcome resistance to MAPKi.

## 2. Results

### 2.1. Preparation of LNPs Encapsulating miRNAs

LNPs encapsulating miR-204-5p, miR-199b-5p or both miRNAs were prepared and characterized as described in the Materials and Methods’ section. The results are summarized in Table 1. The size of empty LNPs was 138.5 nm; the encapsulation of miRNAs led to an increase of LNP mean diameter, ranging from 157.5 to 166.8 nm, independent of the miRNA encapsulated. All the formulations were characterized by a narrow size distribution (PI < 0.2) and a negative ZP (from about −28 to −18 mV).

LNPs-miRNAs, prepared with a theoretical loading of 200 μg miRNA/mg lipids, showed an actual loading ranging from 185.6 to 198.9 g miRNA/mg lipids, corresponding to a very high encapsulation efficiency always >90%.

### 2.2. LNPs Encapsulating miRNAs Impair Proliferation of Melanoma Cell Lines Sensitive or Rendered Resistant to MAPKi

**Figure 1:**
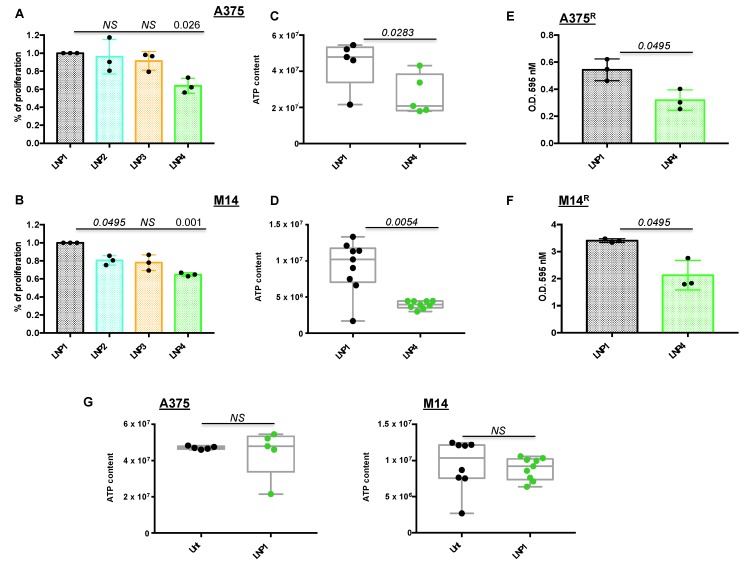
Lipid nanoparticles carrying oncosuppressive miRNAs inhibit melanoma cell proliferation. A375 (**A**) and M14 (**B**) melanoma cells were exposed to 30 μg of the indicated LNPs for 72 h: LNP1 (empty), LNP2 (miR-204-5p), LNP3 (miR-199b-5p) or LNP4 (miR-204-5p + miR-199b-5p). Viability was determined through crystal violet staining to obtain % of cell proliferation as compared to LNP1-treated cells. Cellular ATP was tested by luminescence assay to determine metabolic activity in response to LNP1 and LNP4 treatments in A375 (**C**) and M14 (**D**) cells. Crystal violet staining and O.D. at 595 nM reading by spectrometer assessed the growth inhibitory effects of LNPs in BRAFi-resistant cells (i.e., A375R (**E**) and M14R (**F**)). Cellular ATP measure, performed as described above, was used to test the effects of empty LNPs as compared to untreated A375 and M14 cells ((**G**), left and right panels).

Once LNPs were obtained with optimal technological characteristics in terms of size and miRNA encapsulation efficiency, we started to determine their biological efficacy in two different BRAF-mutant melanoma cell lines in vitro, namely A375 and M14. First of all, we exposed melanoma cells for 72 h to LNP1 (empty), LNP2 (carrying miR-204-5p), LNP3 (carrying miR-199b-5p) or LNP4 (with both therapeutic oncosuppressor miRNAs) to determine their proliferation through crystal violet staining. For these experiments, we used the dose of 30 μg for each LNP, which has been previously reported to be able to efficiently inhibit tumor cell growth via therapeutic nanoparticles [25,28]. Our results demonstrate that only LNP4 is able to significantly impair both A375 and M14 melanoma cell proliferation in vitro (Figure 1A,B), thus suggesting the need to hit multiple oncogenic signaling to exert the maximum therapeutic efficacy. These findings are in line with our previous data [22], and were confirmed also in A375 cells following use of canonical transfection agents of miR-204-5p and miR-199b-5p (Figure S1A).

In order to provide further technical insights into the use of miRNA-carrying LNPs, we conducted proliferation experiments on A375 and M14 cells using different LNP1 and LNP4 concentrations (10, 20, 30 and 40 μg). Results confirm that the best dose to be used in both cell lines is 30 μg because of the highest p-value difference, as compared to the same concentration of LNP1 (Figure S1B). Furthermore, we also exposed A375 cells to LNP1 or LNP4 in the presence or not of fetal bovine serum (FBS), a condition known to be able to hamper the formation of lipoplexes [29]. Results show that the presence of FBS in the culture media does not affect efficient delivery of therapeutic miRNAs to melanoma cells, as clearly demonstrated by qRT-PCR on miR-204-5p and miR-199b-5p expression levels (Figure S2).

Subsequently we determined the effect of nanoparticles on melanoma cells using a variety of biological assays. A375 and M14 cells were treated with LNP1 or LNP4 and tested for the presence of metabolically active and viable cells through ATP quantification. Box plots clearly show that LNPs carrying both therapeutic miR-204-5p and miR-199b-5p are able to reduce cell vitality (Figure 1C,D). Moreover, LNP4 is able to impair the growth of BRAFi-resistant melanoma cells derived from A375 and M14 (A375^R^ and M14^R^, respectively) generated in our laboratory, (Figure 1E,F). Finally, in order to exclude that LNPs growth inhibitory effects is due to the nanoparticles themselves and not to their therapeutic cargo, we exposed melanoma cells to control LNP1. Results, shown in Figure 1G, demonstrated that empty LNPs have no effects on melanoma cell behavior. Of note, the same findings were obtained in a third additional BRAF-mutant melanoma cell line, namely WM266 (Figure S3A). In summary, these results underscore the therapeutic potential of LNPs carrying oncosuppressive miR-204-5p and miR-199b-5p on the inhibition of BRAFi-sensitive and -resistant melanoma cells in vitro.

### 2.3. LNPs Carrying Therapeutic miRNAs Effectively Overexpress miR-204-5p and miR-199b-5p in Melanoma Cells and in Turn Inhibit Their Target Oncogenes Bcl-2 and VEGF-A

**Figure 2:**
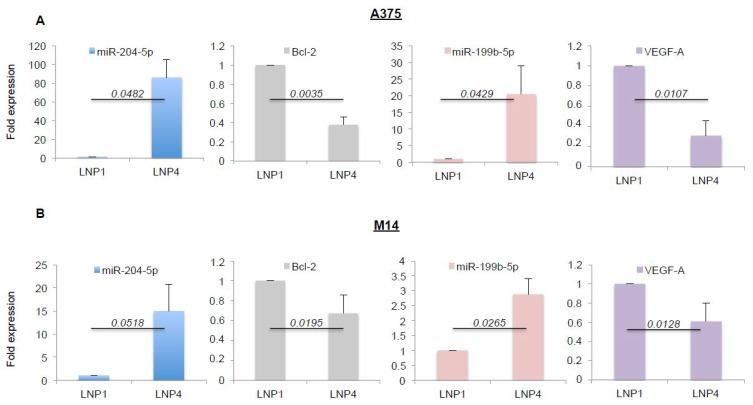
LNP4 upregulates miR-204-5p and miR-199b-5p and inhibits their target genes in human melanoma cells. A375 (**A**) and M14 (**B**) cells were exposed to LNP1 or LNP4, as described above, and then total RNA was extracted to perform qRT-PCR on the expression levels of the indicated miRNAs or their target genes. Results were normalized by ∆∆CT method relative to U6 and GAPDH, respectively.

Given the antitumor efficacy of miRNA-loaded nanoparticles on melanoma cell growth in vitro, we next assessed the effective overexpression of the oncosuppressive miRNAs and the inhibition of some of their target genes. Again, we exposed A375 and M14 melanoma cells to LNP1 or LNP4 for 72 h. Cells were harvested and total RNA was extracted. Results obtained through qRT-PCR demonstrated LNP4 ability to induce a strong increase of the intracellular expression of miR-204-5p (blue bars) and of miR-199b-5p (pink bars), both in A375 and M14 cells (Figure 2A,B respectively). Of note, the same results were observed in WM266 cells (Figure S3B).

**Figure 3:**
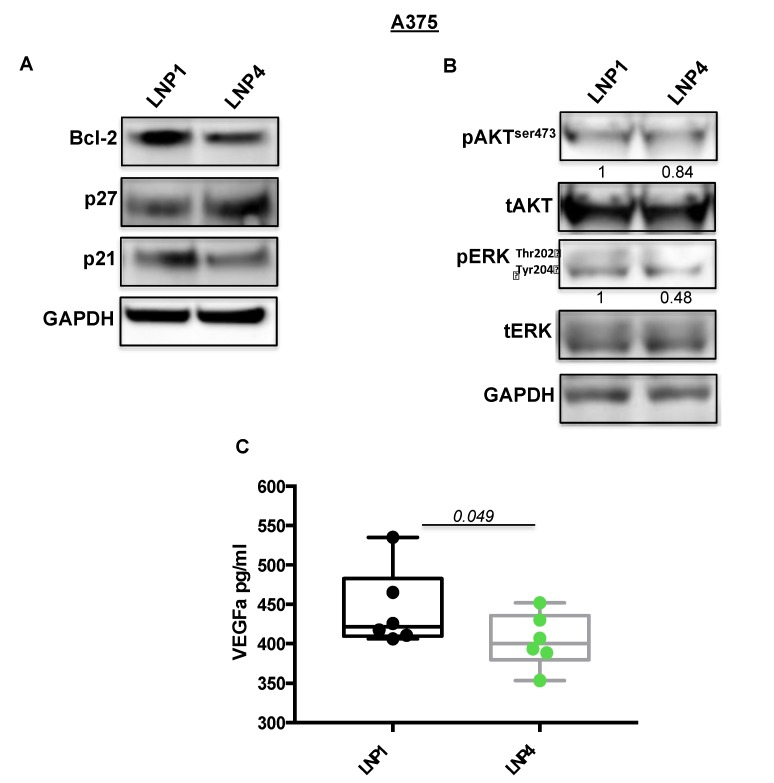
LNP4 affects different downstream effectors in response to the upregulation of miR-204-5p and miR-199b-5p. (**A**,**B**) A375 cells were treated with LNP1 or LNP4, as described above, and total protein extracts were subjected to Western blot analysis to measure the expression levels of the indicated molecular effectors downstream of miR-204-5p and miR-199b-5p. (**C**) ELISA-based reading was used to determine the levels of soluble VEGF-A in response to LNPs treatments in A375 cells.

Subsequently we tested the expression levels of some of their known target genes, namely Bcl-2 for miR-204-5p and VEGF-A for miR-199b-5p [22,30,31]. In line with previous findings, we observed a strong downregulation of the corresponding mRNAs (Figure 2A,B, grey and purple bars), thus supporting the rationale that the inhibitory effects observed in Figure 1 may be caused by the simultaneous hit of multiple oncogenic signaling. In order to confirm this finding we also determined the effects of miRNA carrying LNPs on the protein levels of the same two targets (Figure 3A). Results confirm the ability of LNP4 to inhibit Bcl-2. Furthermore, we also observed LNPs effects on two known cell cycle regulators, namely the cyclins p27 and p21. We observed that miR-204-5p upregulation enhanced p27 mRNA stability: an event reported to occur through the inhibition of a negative regulator of this cyclin, namely Brd4 oncogene [32]. Differently p21 downregulation may be explained by LNPs-induced reduction of the MAPK signaling which is known to induce p21 mRNA transcription [33]. Those latter aspects need further investigation. In addition, we also observed the inhibition of pERK signaling (Figure 3B). Finally, we also evaluated, through quantitative colorimetric ELISA assays, the presence of released VEGF-A factor in culture media following LNPs treatments. Our results clearly indicated a reduction of this pro-angiogenic factor in A375-derived media exposed to LNP4, as compared to that treated with LNP1 (Figure 3C). Of note, those results were confirmed in additional melanoma cell lines, namely M14 and WM266 (Figure S4A–C). Whole blots are available as Figures S5 and S6.

All together, those results indicated that the growth inhibitory effects of LNP4 is mediated by the simultaneous inhibition of Bcl-2 and VEGF-A oncogenes in BRAF-mutant melanoma cells.

### 2.4. Therapeutic LNPs Potentiate MAPKi Action on BRAF-Mutant Melanoma Cell Growth

**Figure 4:**
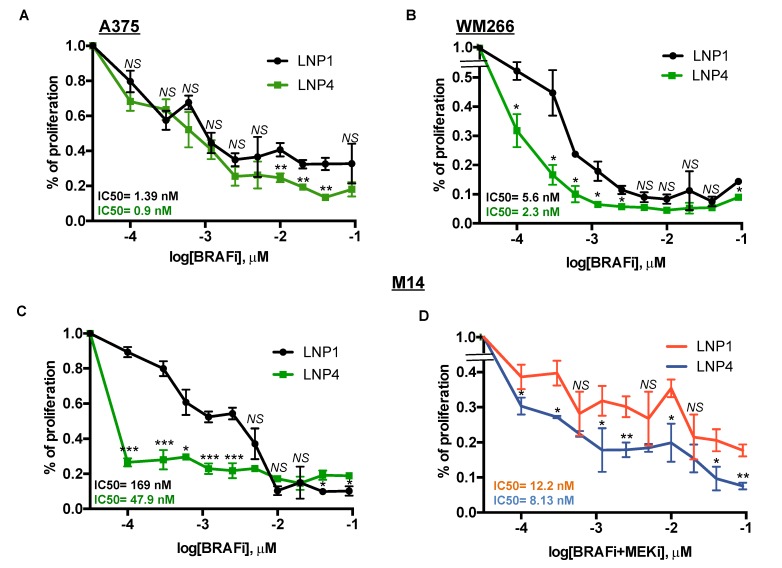
LNP4 potentiates MAPKi growth inhibitory effects on human melanoma cells. A375 (**A**), WM266 (**B**) and M14 (**C**) cells were exposed to dabrafenib in the presence of LNP1 or LNP4 to measure cell viability through ATP reading. LNPs were used at fixed concentration of 30 μg, whereas BRAFi starting from 3 μM and then diluted 1:2 for ten times. (**D**) M14 cells were tested for the combination of dabrafenib + trametinib + LNPs. LNPs and BRAFi were used as described above, whereas MEKi starting from 1.5 μM and then diluted 1:2 for ten times.

Here we sought to determine whether oncosuppressor miRNAs carrying LNPs could be able to potentiate the growth inhibitory effects of MAPKi in BRAF-mutant melanoma cells. For this purpose, we tested three different melanoma cell lines with different degrees of sensitivity to BRAFi according to our previously published data [18,19]. In detail, two of them, namely A375 and WM266, are highly sensitive (IC50 < 10 nM to dabrafenib) to the drug, whereas a third line, i.e., M14 is intrinsically less sensitive (IC50 > 100 nM to dabrafenib). Melanoma cell lines were treated with LNP1 or LNP4 in the presence or not of different concentration of dabrafenib as a BRAFi and cell viability was measured. Results clearly show that the LNP4 carrying oncosuppressive miR-204-5p and miR-199b-5p is able to potentiate BRAFi activity on all three melanoma cell lines (Figure 4A–C). It is important to point out that the best combinatorial effect is observed in the poorly sensitive M14 cells (Figure 4C). Finally, we also tested the triple combination of BRAFi + MEKi + LNPs in M14 cells. Pleasingly we observed also in this case a strong improvement of the growth inhibitory effects in the presence of LNP4 (Figure 4D). Those findings suggest the possibility that nanoparticles carrying the combination of oncosuppressor miRNAs may potentiate the efficacy of current targeted therapies for BRAF-mutant melanoma patients and may delay the emergence of drug resistance.

## 3. Discussion

Target therapy with combination of a BRAF and a MEK inhibitor has dramatically improved the clinical perspective of BRAF-mutated melanoma patients [34]. However, this type of approach still bears a high rate of failures either due to ab initio drug resistance or to acquired resistance, since patients that show initial disease control relapse after a variable period of time. Hence a major medical need is still the development of new combinatorial approaches able to increase the proportion or prolong the duration of therapeutic responses. We believe that therapeutic miRNAs may represent promising candidates in this challenge for their therapeutic potential [12,35]. This assumption arises from our and of others latest works, which underscore the role of those small non-coding RNAs as major modulators of MAPKi resistance in melanoma [21,22,36,37]. In the present work we start to explore the therapeutic potential of two oncosuppressive miRNAs, namely miR-204-5p and miR-199b-5p, which we previously demonstrated to antagonize this process [22]. Oncosuppressive miRNAs cannot be efficiently delivered as naked molecules to cancers in vivo because they are rapidly degraded by serum proteases and do not reach therapeutic concentrations in tumor masses if injected systemically. While cationic liposomes (e.g., lipofectamine) are largely and successfully used in vitro, this system cannot be proposed for systemic administration of miRNAs for future therapies. Indeed, lipoplexes administration by i.v. injection lead to their rapid clearance of aggregation in the bloodstream, with consequent accumulation firstly in the lungs and then in the liver [38]. Thus, in this study, we used LNPs for which the cationic charge is neutralized upon RNA encapsulation, making these nanovectors stable in vivo. In addition, LNPs used in this study are also characterized by a PEGylated lipid that is not present in the lipofectamine and that makes the nanoparticles stealth in the blood circulation. Hence we have decided to adopt a well-characterized delivery approach consisting in the formulation of microRNAs into lipid nanoparticles. This technology has previously shown to allow the efficient delivery of RNA cargo to tumor in mouse tumor models in vivo, and is under advanced clinical development with a product recently approved [27]. In this study we focus on the biophysical and biological characterization of these particles encapsulating miR-204-5p and miR-199b-5p for their therapeutic potential in the treatment of BRAF-mutated melanoma. LNPs offer the ability to encapsulate a high percentage of RNA due to the presence of an ionizable lipid. Indeed, independent of the miRNA used, the encapsulation efficiency was close to 100% of the initially-loaded miRNA. It is worthy of note that, in the case of LNP4, a mix of different miRNA sequences (50% miR-204-5p and 50% miR-199b-5p) was encapsulated, maintaining a very high miRNA loading. On the other hand, the mean size of LNPs lower than 200 nm, as well as their homogeneous size distribution (polydispersity index lower than 0.2 in all the formulations), make these LNP-encapsulating miRNAs suitable for i.v. administration and tumor targeting [38]. Then we moved to the biological characterization of the LNP on different melanoma cells lines. We demonstrated through the use of a combination of in vitro assays that 1) only the delivery of miRNA combinations instead of single miRNAs via nanoparticles is able to achieve a growth inhibitory effect in a variety of BRAF-mutated melanoma cell lines; 2) dual miRNA-loaded LNPs are able to potentiate the growth inhibitory effect of target therapy. As to the first point, our results indicate that the simultaneous targeting of multiple oncogenic signaling is able to exert the most powerful inhibition of melanoma cell growth. This is supported by the evidence that LNPs carrying both oncosuppressive miR-204-5p and miR-199b-5p are able to hit both Bcl-2 and VEGF-A oncoproteins with a superior effect as compared to LNPs carrying only one miRNA. These findings were confirmed also on two melanoma cell lines rendered resistant to a BRAFi. Furthermore LNPs loaded with oncosuppressor miRNAs loaded are able to improve the growth inhibitory power of dabrafenib (BRAFi) on different melanoma cell lines characterized by increasing states of sensitivity to this drug. Furthermore, we show that miR-204-5p and miR-199b-5p are able to potentiate also the combination of a BRAF and a MEK inhibitor, which currently represent the gold standard of therapy for BRAF mutant melanoma patients. Most importantly highest degree of potentiation is observed in the least sensitive of the three melanoma cell lines tested, which suggests that our approach is potentially most helpful in conditions where melanomas are intrinsically more resistant to therapy. The fixed combination of the two miRNAs in the same nanovector should also facilitate the development of a less complex therapy, where this formulation could be combined to the BRAF and a MEK inhibitors. These in vitro findings encourage further testing of miRNA combinations formulated in LNPs in in vivo mouse xenograft models, where it will be possible to assess the capability of these new triple combination to inhibit long term tumor recurrence.

## 4. Materials and Methods

### 4.1. Materials

1,2-dioleyl-3-dimethylammonium propane (DODAP) and N-palmitoyl-sphingosine-1-{succinyl[methoxy(polyethylene glycol)2000]} (PEG_2000_-Cer_16_) were purchased by Avanti Polar Lipids (Darmstadt, Germany). Distearoylphosphatidylcholine (DSPC) was kindly offered from Lipoid GmbH (Cam, Switzerland). Cholesterol (CHOL), sodium chloride, sodium phosphate, HEPES, citric acid and sodium citrate were purchased by Sigma-Aldrich (St. Louis, MO, USA). Ethanol and other solvents were obtained by Exacta Optech (Modena, Italy).

### 4.2. Preparation of LNPs Encapsulating miRNAs

LNPs formulations, blank or encapsulating miRNAs (LNPs-miRNAs), were prepared by ethanol injection method, as previously reported by Semple et al. with some modifications [26]. Briefly, a lipid stock solution composed of DSPC/CHOL/DODAP/PEG_2000_-Cer_16_ (25/45/20/10 molar ratio) was prepared in ethanol (40% *v*/*v* of total preparation). In a separated tube, an aliquot (0.2 mg) of miR-204-5p, miR-199b-5p or both miRNAs was dissolved in 20 mM citric acid pH 4.0 (60% *v*/*v* of total preparation). The two solutions were warmed for 2–3 min to 65 °C and then the lipid ethanol solution was added to the miRNA solution under stirring. The preparation was sized forcing the passage of the suspension through 200 nm (5 times) and 100 nm (5 times) polycarbonate filters using a thermobarrel extruder (Northern Lipids Inc., Vancouver, BC, Canada) maintained at approximately 65 °C. Therefore, the preparation was dialyzed (3.5 kDa cutoff) against 20 mM citrate buffer at pH 4.0 for approximately 1 h to remove excess of ethanol and against HBS (20 mM HEPES, 145 mM NaCl, pH 7.4) for 12–18 h to remove the citrate buffer and to neutralize the LNP surface. Unencapsulated miRNA was removed by ultracentrifugation at 80,000 rpm for 40 min (Optima Max E, Beckman Coulter, Brea, CA, USA; rotor TLA 120.2). Each formulation was prepared in triplicate and stored at 4 °C before use.

### 4.3. LNPs-miRNAs Characterization, Size and Polydispersity Index

The mean diameter and the size distribution (PI) of LNPs-miRNAs were measured by photon correlation spectroscopy (PCS). Briefly, samples were diluted 1:100 *v*/*v* with 0.22 μm filtered water and analyzed with detector at 90° angle by PCS (N5, Beckman Coulter, Brea, CA, USA). As measure of the particle size distribution, polydispersity index (PI) was used. The results were obtained by the average of the measures on three different batches of the same LNP-RNAs formulation.

### 4.4. Zeta Potential of LNPs

The zeta potential (ZP) of the LNPs formulations was determined using a ZetasizerNano Z (Malvern Instruments, Worcestershire, UK). Samples diluted 1:100 *v*/*v* with water and 0.22 μm filtered were prepared and analyzed. For each LNP formulation, the results were obtained by the average of the measures on three different batches.

### 4.5. Lipid Dosage in LNPs

The amount of phospholipid in the LNPs suspension was determined by the Stewart assay [39]. Briefly, an aliquot of the LNPs suspension was added to a two-phase system, consisting of an aqueous ammonium ferrothiocyanate solution (0.1 N) and chloroform. The concentration of DSPC was obtained by measure of the absorbance at 485 nm into the organic layer with an ultraviolet–visible spectrophotometer (UV VIS 1204; Shimadzu Corporation, Kyoto, Japan). The concentration of the total lipid content was calculated considering a constant ratio between the lipids.

### 4.6. miRNA Encapsulation

The amount of miR-204-5p, miR-199b-5p or both miRNAs encapsulated into the LNPs was measured spectrophotometrically. Briefly, an aliquot of the formulation was dissolved in methanol (1:100 *v*/*v*) and samples were centrifugated for 30 min at 13,000 rpm (MIKRO 20; Hettich, Tuttlingen, Germany).

The supernatant was then analyzed at 260 nm. The amount of miRNA loaded into the nanocarriers was expressed as miRNA encapsulation efficiency (EE%), calculated as % ratio between miRNA actual loading (mg of miRNA/mg of total lipids) and miRNA theoretical loading in formulation. For each formulation, the results were calculated as the mean of the measures obtained in three different batches (n = 3).

### 4.7. Cell Lines and Treatments

Human melanoma cell lines, WM266, M14 and A375 were obtained from the laboratory of Dr. Paolo A. Ascierto at National Cancer Institute of Naples “Fondazione G. Pascale”, Naples, Italy. All these cell lines harbor BRAF-V600 mutations that is V600D for WM266 and V600E for M14 and A375. They all derive from metastatic lesions. In particular, M14 was reported to be established at the University of California Los Angeles (UCLA) from a 33-year-old patient with metastatic melanoma, A375 derived from a 54-year-old female with malignant melanoma, whereas WM266 came from individual lymph-node metastases from a 55-year-old female. BRAFi-resistant counterparts were obtained through sequential increasing treatments of a BRAFi for about two months; for more detail please see our previous work. All melanoma cell lines used were cultured in RPMI supplemented with 10% FBS. Nanoparticles treatments were performed by exposing cells to 30 μg of each LNPs for 72 h in the presence of FBS with the exception of setting experiments shown in Figures S1B and S2. Dabrafenib and trametinib as BRAFi and MEKi, respectively, were obtained by Novartis Farma S.p.A. (Rome, Italy). For biological assays they were used at different concentrations, starting from the highest dose of 3 μM and then diluted 1:2 for ten times.

### 4.8. Antibodies and Reagents

Antibodies for Western blotting (i.e., pAKT and pERK) were purchased from Cell Signaling Technology [40] (Boston, MA, USA). Differently, Bcl-2, p21, p27 and GAPDH were obtained from Santa Cruz Biotechnology (Dallas, TX, USA). Anti-rabbit and anti-mouse were purchased from AbCam (Cambridge, UK) [41]. TaqMan probes for miRNAs and target genes were purchased from Applied Biosystems (Foster City, CA, USA). Human VEGF Quantikine ELISA Kit was obtained from R&D Systems (Minneapolis, MN, USA).

### 4.9. VEGF-A Detection

Soluble VEGF-A following LNPs treatments was determined through ELISA kit according to manufacturer’s instructions [42]. Briefly, 200 μL of each sample was incubated for 2 h at room temperature in the 96-well plate, in which a monoclonal antibody specific for human VEGF-A was pre-coated. After washing a specific enzyme-linked polyclonal antibody was added to the wells and then a substrate solution allowed to assess the amount of VEGF-A bound in the initial step by measuring absorbance at 450 nm into microplate reader.

### 4.10. Western Blot Analysis

Melanoma cells were lysed with RIPA buffer purchased by Sigma-Aldrich (St. Louis, MO, USA) and total proteins were run using Invitrogen Bolt Bis-Tris 4–12% Plus gels precast polyacrylamide gels [43]. Proteins were then transferred using the iBlot^®^ Transfer Stacks in nitrocellulose membranes through the fast iBlot® Dry Blotting System (ThermoFisher Scientific, Foster City, CA, USA). The membranes were blocked with 5% non-fat dry milk in TBS 0.1% Tween 20, and incubated with the different primary antibodies O.N. at 4 °C. GAPDH was used to estimate the protein equal loading.

### 4.11. RNA Extraction and Real-Time

Total RNA was extracted using TRIzol method according to manufacturer’s instruction [44] and quantitated by Qubit Fluorometer [45] (ThermoFisher Scientific, Foster City, CA, USA). Quantitative Real Time-PCR (qRT-PCR) was performed by TaqMan Gene Expression Assays (Catalog number 4444556) (Applied Biosystems, Foster City, CA, USA). Each transcript was measured using the comparative Ct method for relative quantification reference to the amount of a common housekeeping gene (i.e., GAPDH or U6 (for miRNAs)).

### 4.12. Cell Viability

The number of viable melanoma cells was measured by quantification of the ATP present according to CellTiter-Glo^®^ Luminescent Cell Viability assay protocol and colony formation assays were performed as previously described [17].

### 4.13. Statistical Analyses

All results reported in this manuscript are presented as mean values from three independent experiments. Quantitative analysis for curve fitting, box plots and *p*-value estimation (significance *p* < 0.05) were performed by GraphPad Prism 7 (San Diego, CA, USA) [46].

## 5. Patents

International application number: PCT/IT2019/050073, Title: “miRNAs for treatment and in vitro diagnosis of drug resistant tumors”.

## Figures and Tables

**Table 1:** Characteristics of LNP blank (LNP1) or encapsulating miRNA (LNP1, LNP2 and LNP3). For each formulation, size, polydispersity index (PI), zeta potential (ZP), actual loading and encapsulation efficiency (EE%) were calculated as the mean of the measures obtained at least on three different batches.

Formulation	miRNA	Diameter (nm) ± SD	PI ± SD	ZP (mV ± SD)	Actual Loading (μg miRNA/mg Lipids)	EE% ± SD
LNP1	-	138 ± 9	0.19 ± 0.02	−11 ± 1	-	-
LNP2	miR-204-5p	157 ± 3	0.17 ± 0.05	−28 ± 8	186 ± 0.005	93 ± 3
LNP3	miR-199b-5p	160.4 ± 11	0.18 ± 0.08	−18 ± 8	199 ± 0.001	99 ± 1
LNP4	miR-204-5p/199b-5p	167 ± 5	0.15 ± 0.05	−26 ± 9	190 ± 0.009	95 ± 5

## Supplementary Materials

The following are available online at https://www.mdpi.com/1422-0067/21/6/1930/s1, Figure S1: miRNA transfection in A375 cells, LNPs dose finding evaluation, Figure S2: miR-204-5p and miR-199b-5p evaluation through qRT-PCR in the presence or not of FBS, Figure S3: ATP content evaluation in WM266 following LNP exposure and miRNAs measure through qRT-PCR, Figure S4: Western blot on M14 cells following LNP treatments and VEGF-A evaluation in WM266 cells treated as above, Figure S5: Whole blots of Figure 3A, Figure S6: Whole blots of Figure S4A.

## Abbreviations

MAPKi	BRAF/MEK inhibitors
LNP	Lipid nanoparticles
FBS	Fetal bovine serum
