# Supplementary material for: In Vitro Biophysical and Biological Characterization of Lipid Nanoparticles Co-Encapsulating Oncosuppressors miR-199b-5p and miR-204-5p as Potentiators of Target Therapy in Metastatic Melanoma
Source: Int J Mol Sci. 2020 Mar 12;21(6):1930. doi: 10.3390/ijms21061930 (PMC7139872; doi:10.3390/ijms21061930)
Supplement: Supplementary file 1 [file ijms-21-01930-s001.pdf]

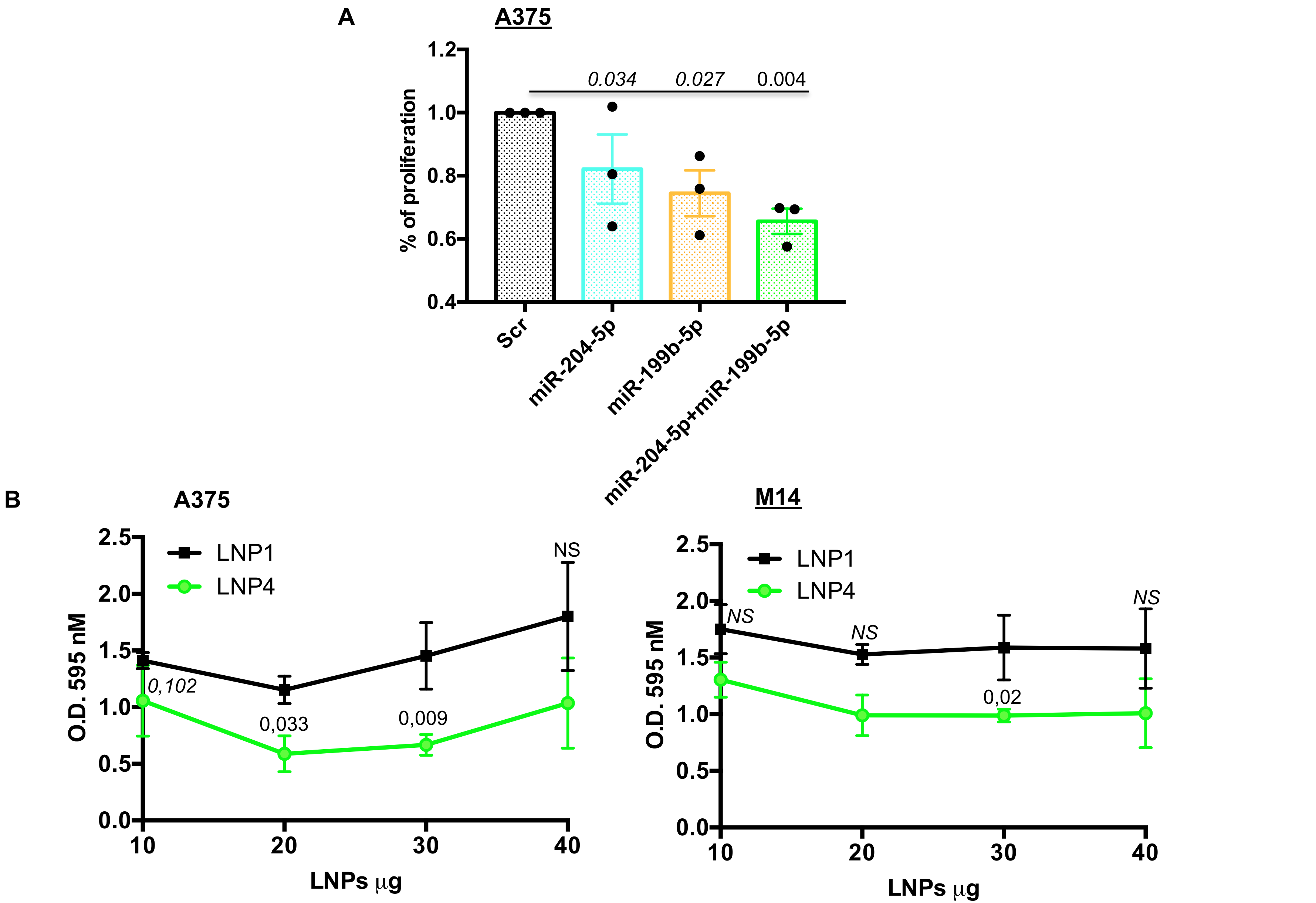

Supplementary Figure 1. miR-204-5p and miR-199b-5p inhibits A375 cell growth and LNPs dose response experiments. (**A**) A375 melanoma cells have been transfected with the indicated miRNAs alone or in combination to determine cell proliferation as compared to scrambled transfected cells. (**B**) A375 (left panel) and M14 (right panel) cells have been exposed to different concentrations of LNP1 or LNP4 to assess the best dose to achieve the maximum growth inhibitory effect through O.D. reading at 595 nM of crystal violet dye.

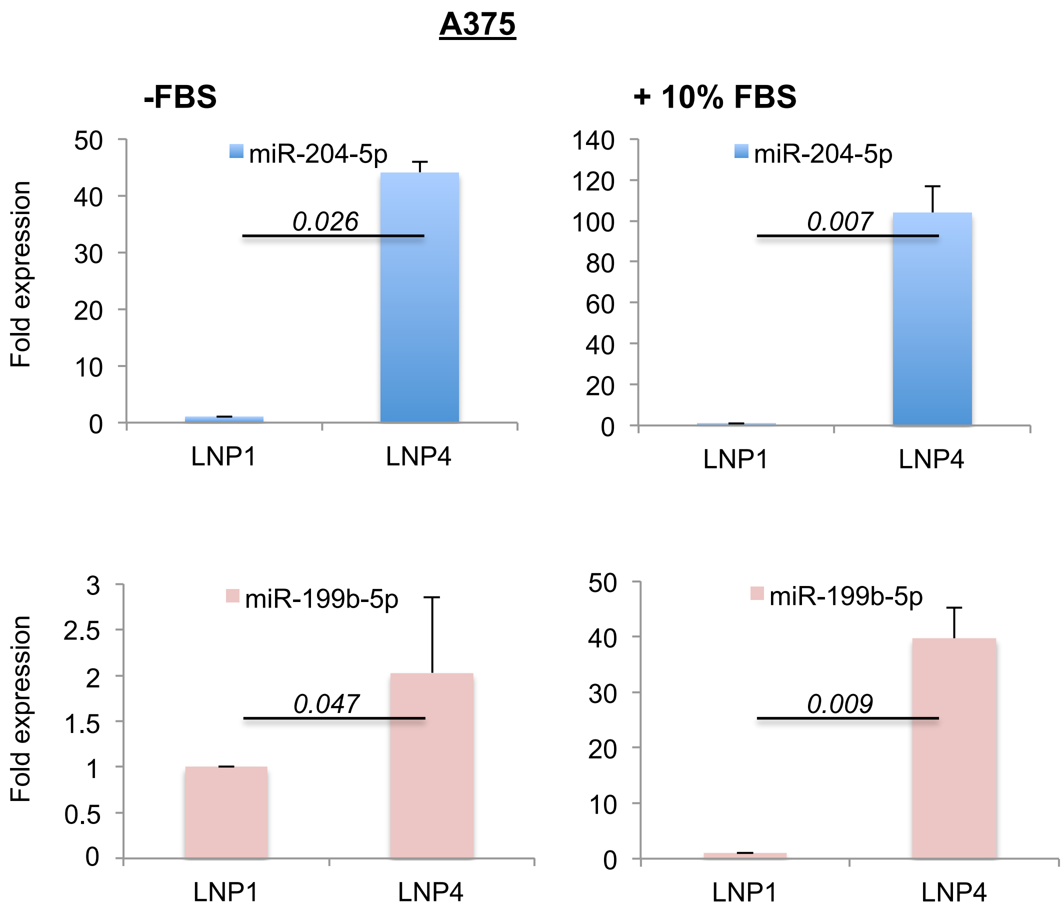

Supplementary Figure 2. FBS presence does not affect LNP4-induced upregulation of miR-204-5p and miR-199b-5p. (**A**) A375 cells have been treated with LNP1 or LNP4 in the absence of FBS (left panels) or in the presence of 10% FBS (right panels) and then total RNA was extracted to perform qRT-PCR on the expression levels of the indicated miRNAs. Results have been normalized by ΔΔCT method relative to U6.

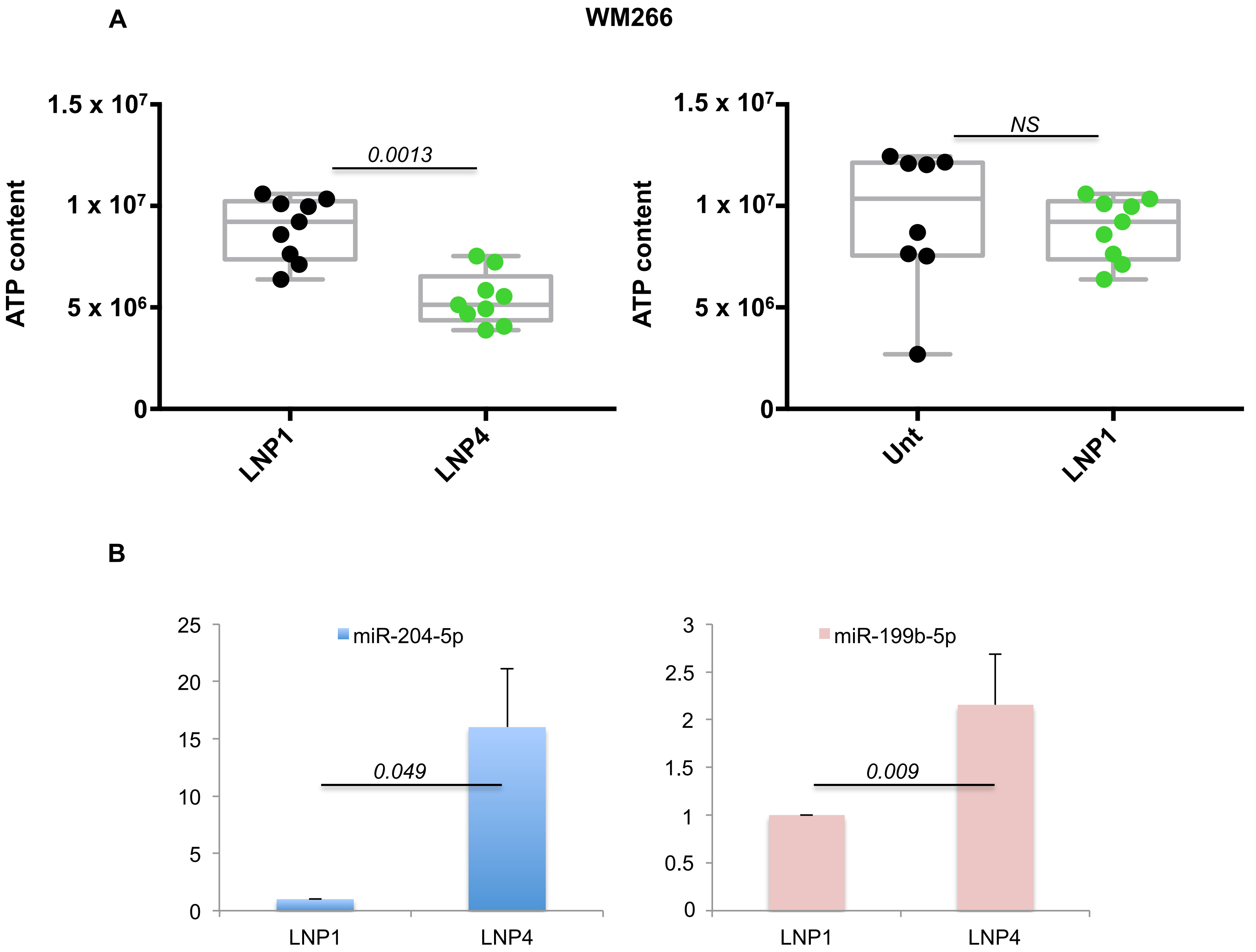

Supplementary Figure 3. Lipid nanoparticles carrying oncosuppressive miRNAs inhibit WM266 melanoma cell proliferation. (**A**) Cellular ATP has been tested by luminescence assay to determine metabolic active cells in response to LNP1 or LNP4 treatments (left panel) or to test the basal toxicity of LNP1 as compared to untreated cells (right panel). (**B**) WM266 cells exposed to LNP1 or LNP4 have been collected to perform qRT-PCR on miR-204-5p and miR-199b-5p expression levels as described above.

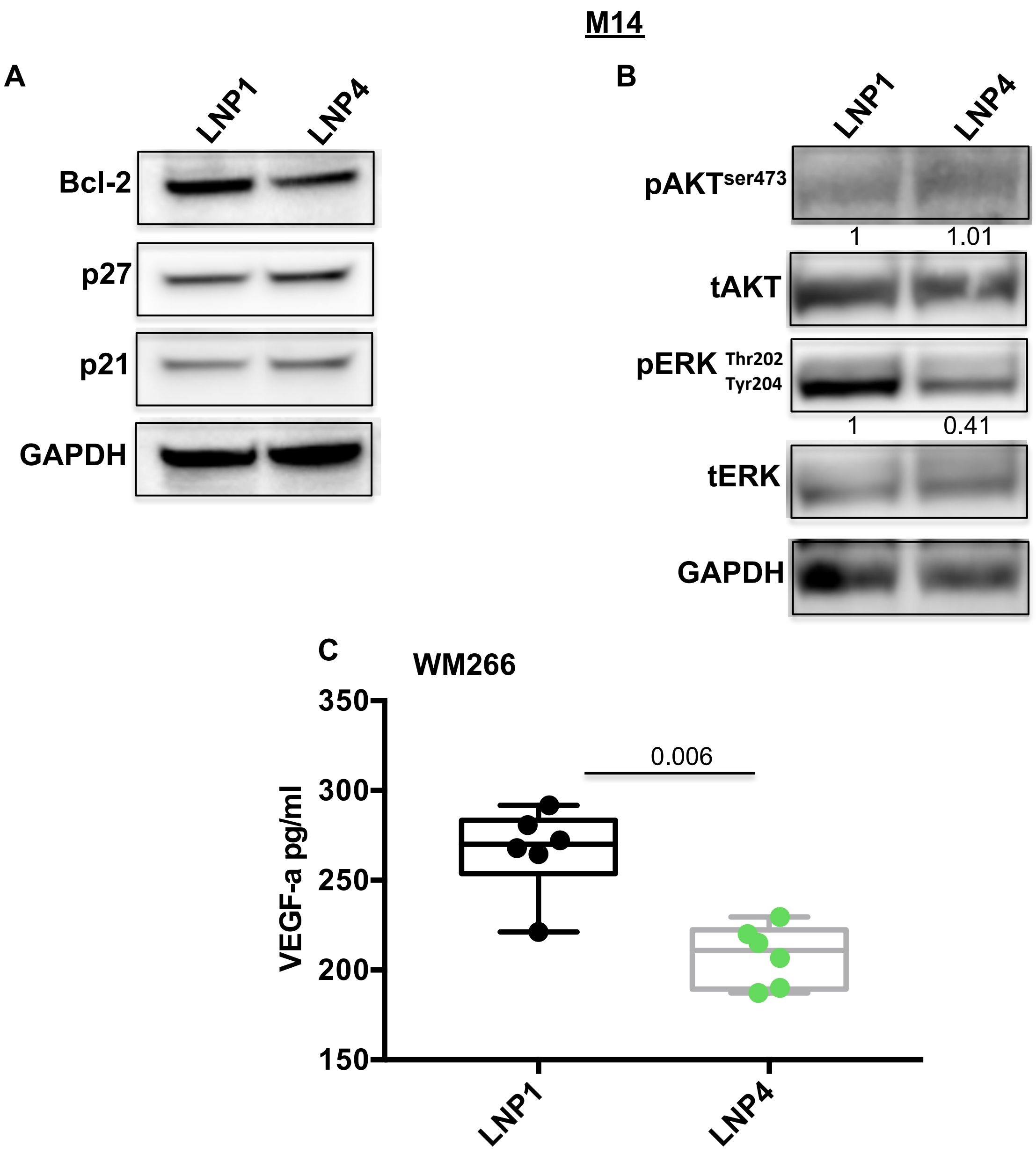

Supplementary Figure 4. LNP4 affects different downstream effectors in human melanoma cells. (**A** and **B**) M14 cells have been treated with LNP1 or LNP4 as described above and total protein extracts have been subjected to Western Blot analysis to measure the expression levels of the indicated molecular effectors downstream of miR-204-5p and miR-199b-5p. (**C**) ELISA-based reading was used to determine the levels of soluble VEGF-A in response to LNPs treatments in WM266 cells.

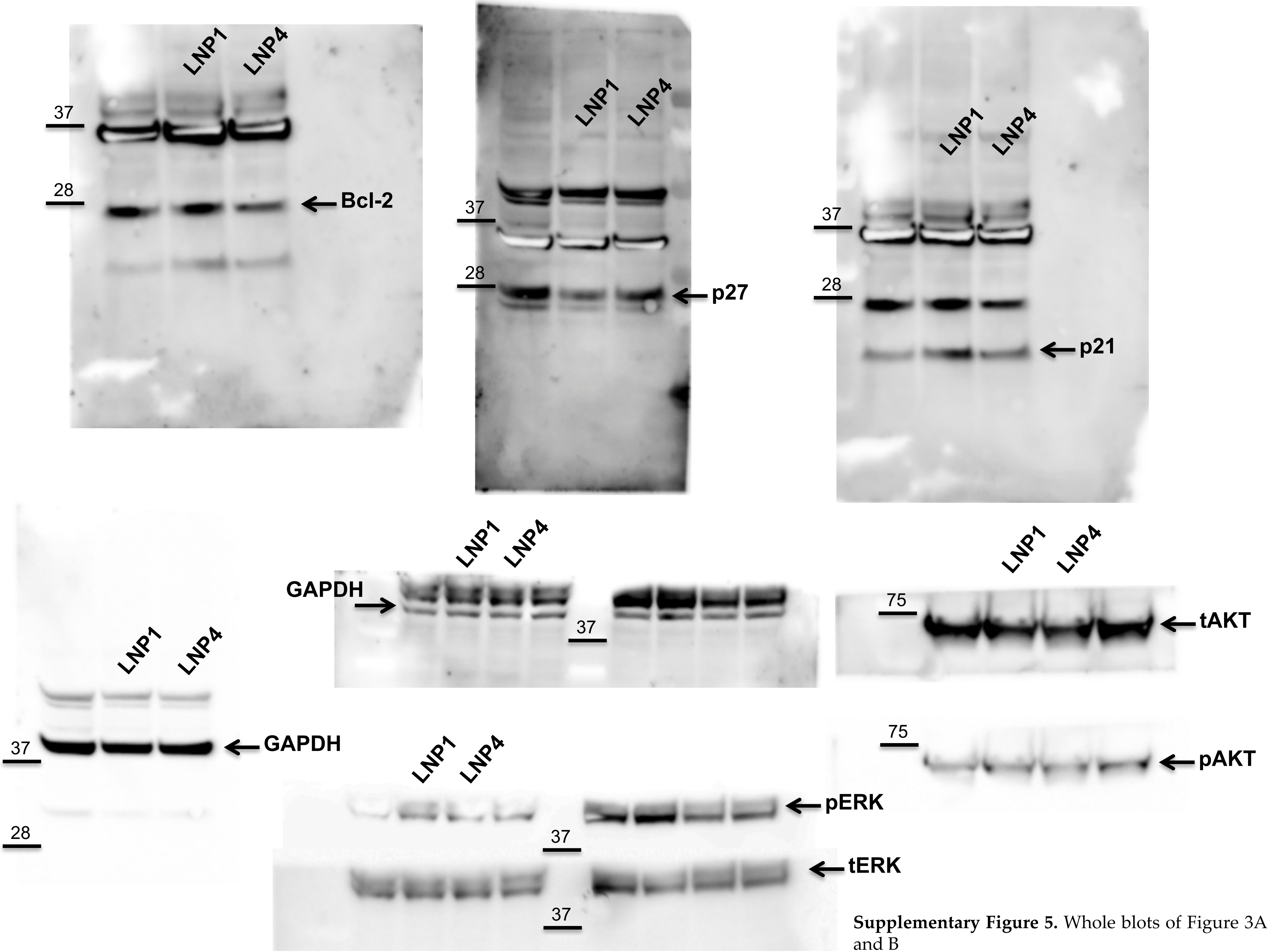

Supplementary Figure 5. Whole blots of Figure 3A and B

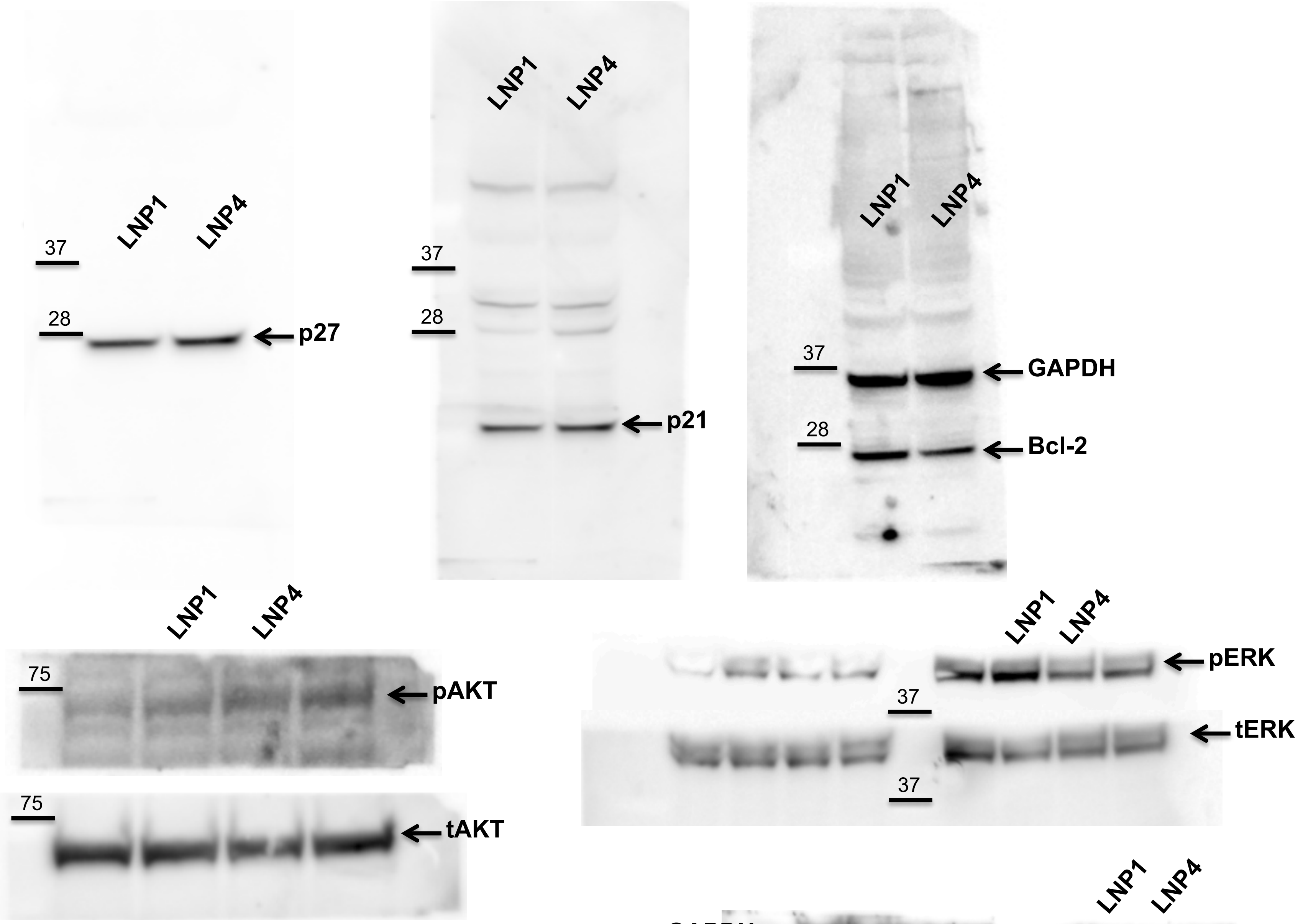

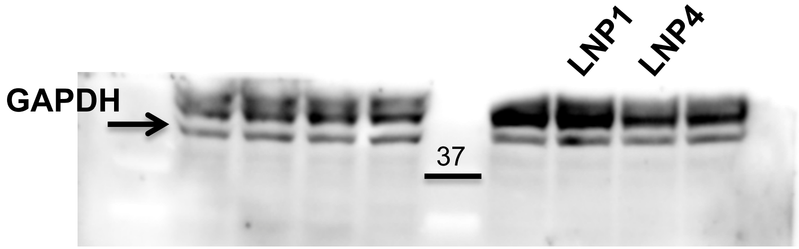

Supplementary Figure 6. Whole blots of Suppl. Figure 4A and B
